# The Impact of Instrument-Specific Musical Training on Rhythm Perception and Production

**DOI:** 10.3389/fpsyg.2016.00069

**Published:** 2016-02-03

**Authors:** Tomas E. Matthews, Joseph N. L. Thibodeau, Brian P. Gunther, Virginia B. Penhune

**Affiliations:** Laboratory for Motor Learning and Neural Plasticity, Department of Psychology, Concordia UniversityMontreal, QC, Canada

**Keywords:** rhythm perception, rhythm production, beat perception, musical training, motor timing, expertise, tapping

## Abstract

Studies comparing musicians and non-musicians have shown that musical training can improve rhythmic perception and production. These findings tell us that training can result in rhythm processing advantages, but they do not tell us whether practicing a particular instrument could lead to specific effects on rhythm perception or production. The current study used a battery of four rhythm perception and production tasks that were designed to test both higher- and lower-level aspects of rhythm processing. Four groups of musicians (drummers, singers, pianists, string players) and a control group of non-musicians were tested. Within-task differences in performance showed that factors such as meter, metrical complexity, tempo, and beat phase significantly affected the ability to perceive and synchronize taps to a rhythm or beat. Musicians showed better performance on all rhythm tasks compared to non-musicians. Interestingly, our results revealed no significant differences between musician groups for the vast majority of task measures. This was despite the fact that all musicians were selected to have the majority of their training on the target instrument, had on average more than 10 years of experience on their instrument, and were currently practicing. These results suggest that general musical experience is more important than specialized musical experience with regards to perception and production of rhythms.

## Introduction

Perceptually grouping a series of auditory events into a coherent rhythmic pattern within the context of music is a skill that develops early and is likely innate, at least in humans (Phillips-Silver and Trainor, 2005; Iversen et al., 2008; Honing, 2012). Production of a musical rhythm involves the temporally precise coordination of auditory and motor processes at a level not seen in other domains (Zatorre et al., 2007). Perception and production of rhythms are likely universal skills as most people can synchronize their movements to music, even without any formal training. However, various studies have shown that musical training can improve rhythmic perception and production (Smith, 1983; Drake, 1993; Kincaid et al., 2002; Chen et al., 2008), fine-grained temporal processing (Drake and Botte, 1993; Rammsayer and Altenmüller, 2006; Repp, 2010; Farrugia et al., 2012; van Vugt and Tillmann, 2014) and precise motor synchronization (Franek et al., 1991; Collier and Ogden, 2004; Repp and Doggett, 2007; Repp, 2010; Baer et al., 2013, 2015). These improvements are hypothesized to be driven by reinforced connections between sensory, proprioceptive, cognitive, and motor systems resulting from years of instrumental practice (Herholz and Zatorre, 2012).

These findings tell us that music training can result in rhythm processing advantages, but they do not tell us whether practicing a particular instrument could lead to specific effects on rhythm perception or production. We might hypothesize that those musicians whose training emphasizes rhythm, or whose instrument requires motor skills similar to those used in tests of rhythm production, would out-perform musicians whose training emphasized pitch or whose instrument requires very different effectors. There are many studies showing differences between musicians and non-musicians (e.g., Chen et al., 2008; Repp, 2010), however, there are very few studies that have looked at the effects of specialized training on rhythm processing among different groups of musicians (e.g., Krause et al., 2010; Cicchini et al., 2012). These studies, which are reviewed below, suggest that subtle differences may exist between musician groups, but the findings are inconsistent. Most studies assessed relatively low-level aspects of rhythm processing, such as timing sensitivity and isochronous tapping rather than higher-level aspects, such as beat perception or rhythm synchronization. In addition, none assessed possible systematic differences in the duration and type of musical training across instrument types. To address these issues, the current study compared four groups of musicians: drummers, singers, pianists, and string players as well as a control group of non-musicians on a battery of rhythm perception and production tasks. We predicted that drummers, whose training focuses on rhythm processing, and pianists, whose motor skills match the demands of rhythm production tasks might perform better than violinists and singers, whose training emphasizes pitch processing and whose motor skills require different effectors and movements.

Many researchers have tested the effects of musical training on rhythm processing by comparing musicians and non-musicians. These studies can be divided into two main categories, those that focus on higher-level processing of meter and beat, and those concerned with lower-level motor and timing processes. These studies show that musical training improves rhythm processing in two ways. On the one hand, musical experience improves the ability to use a rhythmic framework to find the underlying pulse and parse the metrical structure (Smith, 1983; Drake, 1993; Chen et al., 2008; Grahn and Rowe, 2009). On the other hand, training improves lower-level abilities, such as fined-grained timing perception, sensorimotor synchronization, and continuation tapping (Franek et al., 1991; Collier and Ogden, 2004; Repp, 2010; Baer et al., 2013, 2015).

High-level rhythm processing was investigated in a functional magnetic resonance imaging (fMRI) study which tested the effects of metrical complexity and musical training using a rhythm synchronization task (RST; Chen et al., 2008). They found that the musicians were more synchronous and less variable than the non-musicians. Further, the musicians reported that they imposed a metrical structure on the rhythms whereas the non-musicians reported a chunking strategy. During the task musicians showed more engagement of right prefrontal brain regions, consistent with the use of a top-down, meter-based strategy. Further support for the idea that training leads musicians to adopt a meter-based strategy in rhythm tasks comes from studies showing improved rhythm reproduction (Smith, 1983; Drake, 1993) as well as differences in brain activity and ratings of beat presence in musicians compared to non-musicians (Grahn and Rowe, 2009).

Other studies have focused on improvements in lower-level timing and motor functions due to musical training. Generally, musicians show more accurate tapping and/or lower tapping variability on synchronization and continuation tapping tasks as well as a timed sequence production task, compared to non-musicians (Drake et al., 2000; Kincaid et al., 2002; Aoki et al., 2005; Repp, 2010; Baer et al., 2013). Furthermore, previous work has shown that musical expertise leads to improvements in tempo sensitivity (Drake and Botte, 1993), sensitivity to phase shifts (Repp, 2010), anisochrony detection (Friberg and Sundberg, 1995) and duration reproduction (Franek et al., 1991).

Recently, two separate groups have developed batteries of rhythm and timing tasks that assess both higher-level and lower-level aspects of rhythm perception and production. The Battery for the Assessment of Auditory Sensorimotor and Timing Abilities (BAASTA; Farrugia et al., 2012) includes four timing and rhythm perception tasks and four tapping tasks. The authors found that musicians were better than non-musicians on all four perceptual tasks which tested both high-level and low-level processing. Only non-musicians were tested on the tapping tasks. The Harvard Beat Assessment Test (H-BAT; Fujii and Schlaug, 2013) includes four beat-based tasks involving both higher-level and lower-level processing and both perceptual and productive components. Although musicians and non-musicians were not compared explicitly, training was significantly correlated with measures of synchronization consistency.

Taken together, studies comparing musicians and non-musicians show that training improves performance on both higher- and lower-level aspects of rhythm processing. But, they do not tell us about the possible effects of training on a particular instrument.

Several studies comparing rhythm experts (i.e., drummers and percussionists) to other musician and non-musician groups have shown that rhythm training generalizes to low-level timing abilities. For example, percussionists detected smaller timing deviations in a discrimination task compared to classical musicians (three pianists and one singer) and non-musicians, who did not differ (Ehrlé and Samson, 2005). Two other studies compared musician groups on low-level production tasks with both auditory and visual stimuli. The first tested drummers, string players and non-musicians on an interval reproduction task. Drummers were less variable and used a different strategy than the other groups on the visual task but not the auditory task (Cicchini et al., 2012). The second tested drummers, professional pianists, amateur pianists, singers, and non-musicians on a production task where participants synchronized taps to an isochronous signal (Krause et al., 2010). The professional pianists had approximately 25 years of experience with their instrument on average while the amateur pianists and drummers had approximately 15 years of experience on average. The singer group had approximately 22 years of singing experience on average. During auditory synchronization, drummers had significantly smaller asynchronies than the amateur pianists, and were less variable in their tapping compared to singers, amateur pianists, and non-musicians. However, there was no difference between the drummers and professional pianists. Two other studies found that experienced drummers had reduced tapping variability compared to non-drummers, however, it was not clear if the non-drummers played other instruments (Fujii and Oda, 2006; Fujii et al., 2009). Non-drummer musicians, specifically brass-players and pianists, have also shown enhanced performance compared to non-musicians on low-level perceptual and production tasks (van Vugt and Tillmann, 2014). Unexpectedly, there were no differences in performance between the two musician groups. The authors determined that individual differences in synchronization ability were better predictors of performance on the perceptual tasks, over and above musical experience. This suggests that musical training improves synchronization abilities which generalize to better timing perception, regardless of the instrument-specific experience.

Rhythm experts have also shown enhanced performance on higher-level rhythm tasks. These tasks test one’s ability to perceive and synchronize to metrical structures rather than testing timing sensitivity or synchronization to an isochronous sequence. One study compared a ‘rhythm expert group,’ consisting of four percussionists and one violinist who had performed particularly well in previous tapping experiments, to other well-trained musicians on a bimanual Synchronization–Continuation task using non-isochronous rhythms (Repp et al., 2013). This ‘rhythm expert group’ was less variable in their tapping than the other musicians for the faster rhythms only, suggesting a context-dependent effect. Another study showed that percussionists performed better on beat tapping and rhythm reproduction tasks compared to a non-percussionist group that included both musicians and non-musicians (Cameron and Grahn, 2014). Finally, a recent study showed that percussionists were better at a beat-based perceptual timing task compared to non-percussionists when synchronizing to the beat (Manning and Schutz, 2015). However, no differences were seen between groups in the no-movement condition, suggesting that motor synchronization is crucial for the beat-based timing benefits that comes with rhythm-focused training. It should be noted that in the latter two studies the musicians in the non-percussionist group were not explicitly identified, therefore conclusions cannot be made regarding the effects of specialized instrumental training.

Together, these studies suggest that drummers and percussionists may have superior low-level timing abilities. However, the results are not always consistent. Krause et al. (2010) showed that professional drummers showed enhanced synchronization abilities compared to amateur pianists and non-musicians, but not compared to professional pianists. Other studies showed enhanced performance in drummers over non-drummers but the non-drummer groups were not well defined (Fujii and Oda, 2006; Fujii et al., 2009). Further, many of these studies did not quantify the length and type of musical experience in their musician groups. Thus, it is difficult to ascertain whether it is the amount or the type of musical training that determines low-level timing ability. Only two studies have looked at higher-level rhythmic abilities among different musician groups (Repp et al., 2013; Cameron and Grahn, 2014) and they do not lead to strong conclusions regarding the effect of specialized musical training. Therefore, the current study used a battery of four rhythm perception and production tasks that were designed to test both higher- and lower-level aspects of rhythm processing. In order to address effects of training, participants were selected to have similar levels of experience with their primary instrument. Furthermore, measures related to the amount of training were collected in order to compare across groups.

In choosing which tasks to include in our battery, we focused on the two key processes involved in perceiving and producing a musical rhythm. First, the underlying beat or pulse is extracted by finding the most stable and/or salient isochronous structure within a rhythm. Secondly, elements of the rhythm are grouped into hierarchical (i.e., metrical) structures based on explicit and subjective accents as well as one’s knowledge of musical patterns (Fitch, 2013). To test these higher-level processes we used the RST, the Beat Synchronization Task (BST) and a perceptual version of the Beat Alignment Test. In the RST, participants were asked to listen to rhythms and then synchronize finger taps with each note of the rhythm upon the second presentation (Chen et al., 2008). In the BST, participants were asked to synchronize finger taps with the underlying beat of a rhythm during the second and third presentations of that rhythm (Kung et al., 2013). Separate rhythms were used in the Beat and RSTs. The Beat Alignment Test involves judging whether a metronome was synchronized with the underlying beat of musical excerpts (Iversen and Patel, 2008). A variant of the Synchronization–Continuation Task (SCT) was used to test lower-level, motor timing processes required to synchronize one’s movements to the underlying beat or individual onsets of a rhythm (Stevens, 1886). In this task participants synchronized their taps to a metronome and then continued tapping at the same rate as accurately as possible after the metronome had stopped.

Auditory short term memory (including working memory) has been correlated with rhythm abilities generally (Grahn and Schuit, 2012) and performance on the RST specifically (Bailey and Penhune, 2012). Therefore, two measures of auditory working memory [the Digit Span (DS) and Letter Number Sequencing (LNS) tasks from the WAIS-IV; Wechsler, 2008] were included to investigate whether differences in rhythm abilities among groups were related to auditory working memory.

The goal of this study was to investigate the effects of specialized music training on rhythm perception and production by comparing four musician groups (drummers, pianists, string players, and singers) and a control group of non-musicians on a battery of rhythm tasks. These tasks involved both higher-level cognitive and low-level motor timing aspects of rhythm processing in order to investigate whether these processes are affected differentially by specialized musical training. Differences between groups were expected as the musicians differed in terms of their rhythm expertise and the match between the motor skills required for their instrument and those required for the tasks.

## Materials and Methods

### Experimental Design

All participants performed the BST, the RST, the Synchronization–Continuation task, the beat alignment perception task (BAPT) and two working memory tasks (DS and LNS). As discussed above, the four rhythm tasks were included to test four different aspects of rhythm processing including basic abilities, such as sensorimotor synchronization and higher level abilities, such as beat and meter perception and production. The tasks were administered in a counter-balanced order, except that the RST and BST were never administered consecutively. DS and LNS were administered during the break between the two blocks of the RST and BST with the DS always administered first. The whole battery of tasks took approximately 2 h. Stimuli were presented and responses recorded on an IBM-compatible computer. All stimuli were played through Sony MDR 7506 headphones at a comfortable volume.

### Participants

We tested forty-two musicians (9 drummers, 11 pianists, 10 singers, and 12 string players) and 14 non-musicians (age 18 to 35 [*M* = 23.54, *SD* = 4.46]). Musicians were selected to have at least 10 years of experience on their primary instrument and to have limited training with any of the other target instruments or singing. The non-musician group included individuals with less than 3 years of musical training or experience, who did not have any formal training in the last 5 years and were not playing an instrument at the time of testing. Participants completed an extensive Musical Experience Questionnaire (MEQ) developed in our laboratory (Bailey and Penhune, 2010). From this questionnaire, we extracted five variables that we thought were most important in characterizing the groups: age of start, number of years playing primary instrument, number of years playing any instrument, current hours of practice per week and years of lessons (see Results and **Table 1** for a detailed report of these measures).

**Table 1:** Descriptive measures and musical experience.

	Overall (*N* = 56)	Drummers (*N* = 9)	Pianists (*N* = 11)	Singers (*N* = 10)	Strings (*N* = 12)	Non-musicians (*N* = 14)
Age	23.54 (4.46)	23.00 (4.24)	23.45 (4.5)	24.80 (6.01)	22.33 (3.55)	24.07 (4.30)
Sex (% female)	63%	0	55%	80%	75%	86%
Age of start	9.29 (3.46)	11.2 (3.27)	7.6 (2.12)	10.7 (4.32)	8.08 (2.78)	11.04 (5.41)
Years of lessons (primary)	10.17 (5.88)	5.44 (3.61)	12.9 (2.96)	11.3 (7.97)	10.5 (5.53)	0.96 (1.04)
Years playing						
Primary	13.76 (5.2)	11.89 (5.37)	15.1 (3.48)	13.7 (7.15)	14.08 (4.60)	1.11 (1.04)
Secondary	2.76 (2.41)	3.43 (2.51)	2.63 (2.38)	3.22 (2.28)	2.3 (2.87)	
Current practice (Hours per week)						
Primary	10.7 (12.20)	14.06 (13.29)	15.5 (16.00)	6.1 (5.84)	8.00 (10.89)	
Secondary	1.76 (3.29)	2.3 (2.86)	3.80 (6.50)	0.46 (0.77)	2 (2.07)	

*Values outside parentheses are means and values inside parentheses are standard deviations.*

Participants were recruited, via advertisements placed online and around the McGill and Concordia University campuses. All were right-handed, free of any neurological disorders and reported no motor or hearing problems. Written informed consent was obtained in accordance with the Declaration of Helsinki and participants were compensated for their time. The study was approved by the Concordia University Human Research Ethics Committee.

### Test Battery

#### Rhythm Synchronization Task (RST)

##### Stimuli and Procedures

The RST is a variant of the task developed by Chen et al. (2008) and has been used in several previous studies with musicians and non-musicians (Bailey and Penhune, 2010, 2012). The RST requires participants to listen to, and then tap in synchrony with a series of auditory rhythms varying in metrical complexity. Metrical complexity was defined based on the rules of metric organization of Povel and Essens (1985), who showed that as the number of sounds that fall on predicted beat points increases, the metrical strength of the rhythm increases. All rhythms were comprised of the same 11 woodblock notes: five eighth notes (250 ms), three quarter notes (500 ms), one dotted quarter note (750 ms), one half note (1000 ms), and one dotted half note (1500 ms). The duration of the woodblock sound was always 200 ms and each rhythm sequence lasted 6 s. These notes were rearranged to create six different rhythms, two at each level of complexity: metrically simple (MS), metrically complex (MC), and non-metric (NM), (see Chen et al., 2008 and Bailey and Penhune, 2010 for a more detailed description of the stimuli). Each trial had two parts: listen and synchronize. During the listen phase, participants listened to each rhythm without moving. During the synchronization phase, participants tapped in synchrony with each note of the rhythms using the right index finger on the computer mouse. The task included two blocks of 36 trials (six repetitions of each rhythm). Between the listen and synchronization phases there was a three second silence followed by a warning sound, followed by an interval of 750 ms. Rhythms were presented and tapping responses were recorded using Presentation, v0.8, (Neurobehavioral Systems).

##### Measures

Rhythm synchronization performance was assessed using two measures: the percentage of correct taps (percent correct) and the inter-tap interval deviation (ITI deviation). A tap was considered correct if it was made within half of the onset-to-onset interval (IOI) before or after each woodblock note. For correct taps only, the ITI deviation was calculated by dividing the ITI by the IOI, subtracting this ratio from one and then taking the absolute value. This measure is indicative of how well participants have reproduced the temporal structure of the rhythms. Both measures were calculated for each trial and averaged across rhythm type and block.

#### Beat Synchronization Task (BST)

##### Stimuli and procedure

The BST was developed in our laboratory as part of an fMRI study (Kung et al., 2013). In this task, participants are required to listen to, and then tap in synchrony with the beat of musical rhythms that vary across four levels of metrical complexity. As in the RST, metrical complexity, or beat strength, was defined based on the rules of metric organization of Povel and Essens (1985).

Rhythms were either in duple (20 rhythms) or triple meter (12 rhythms), and could have two different tempos (16 each). All rhythms were comprised of the same 11 woodblock notes (100 ms). Each of the duple rhythms contained five eighth notes (195 or 260 ms in fast and slow tempi, respectively), three quarter notes (390 or 520 ms), one dotted quarter note (585 or 780 ms), and one half note (780 or 1040 ms). Rhythms at the fast and slow tempi lasted 3.51 and 4.68 s, respectively. Rhythms were divided into four levels of complexity (perfectly metric, strongly metric, metric, and weakly metric) based on the number of notes falling on predicted beats points. For the perfectly metrical rhythms, there were note onsets at all predicted beat points (five or seven onsets on the beat for the duple and triple meters, respectively), whereas the weakly metric rhythms had stimulus onsets at only a subset of the predicted beat points (two or four onsets on the beat for the duple and triple meters, respectively; see Kung et al., 2013 for more detail on how the stimuli were created). *C*-scores were also calculated based on the model of Povel and Essens (1985). A *C*-score is the amount of counterevidence a rhythm supplies regarding the beat locations based on the number of silences and weakly accented notes that fall on predicted beat points (see Povel and Essens, 1985 for more detail on how *C*-scores are calculated). *C*-scores within each level of complexity were highly consistent (see **Table 2**).

**Table 2:** *C*-scores for rhythms in the BST.

	Perfectly metric	Strongly metric	Metric	Weakly metric
Quadruple (Duple)	0–1 (8–9)	4–5 (12–13)	8–9 (17)	12–14 (21–23)
Triple	0	5	9	13

Each trial contained three repetitions of each rhythm. During the first repetition participants were instructed to listen and find the beat of the rhythm without moving. During the next two repetitions, which were interleaved with warning sounds, participants were instructed to tap in synchrony with the beat of the rhythms using the right index finger on the computer mouse. The intervals preceding and following the warning sounds were twice the length of the IBI so as not to interfere with the pulse of the rhythm. Participants were not instructed as to what beat level they should tap for each rhythm (i.e., quadruple, duple, sextuple, or triple). The order of trials in triple or duple meter, and fast or slow tempi, was pseudo-randomized to prevent participants from carrying over the beat from one trial to the next. The task was split into two 11 min blocks, each consisting of 32 trials, where each rhythm was used once per block. The rhythms in the two blocks were alternated such that the slow rhythms in the first block were the fast rhythms in the second. Rhythms were presented and tapping responses were recorded using Presentation, v0.8, (Neurobehavioral Systems).

##### Measures

Beat synchronization performance was assessed by calculating the percentage of correct taps (percent correct) and the ITI deviation. A tap was considered correct if it was made within 20% of the inter-beat interval (IBI). A lower percentage was used in this task compared to the RST as it was a percentage of the single-length IBI rather than the multiple-length IOIs of the RST sequences. Participants were not instructed as to what metric level they were to tap, therefore the first step in the analysis was to determine whether they tapped at the duple or quadruple level, thus determining the target IBI. This was done by inspecting the tap data, comparing average ITIs to target IBIs and comparing the number of taps to the expected number of taps. If the average ITI for a particular trial was greater than the target ITI plus 50% it was determined that the participant tapped at the quadruple level rather than the duple. Similarly to the RST, ITI deviation was calculated, however, for the BST, the ratio of the ITI and IBI was calculated rather than the ratio of ITI and IOI. Percent correct and ITI deviation were calculated for each trial and averaged across meter, tempo, and metrical complexity.

#### Synchronization–Continuation Task (SCT)

##### Stimuli and procedure

This is a variant on the commonly used synchronization–continuation task (Repp, 2005) which has been used in our lab previously to measure self-paced isochronous tapping (Baer et al., 2013, 2015). We used the identical experimental setup, data cleaning and analysis procedures as those used by Baer et al. (2015). In the paced phase, participants were asked to tap in synchrony with a metronome (1 KHz pure tone, 20 ms in duration; 35 cycles). In the continuation phase after the metronome stopped, participants were instructed to continue tapping at the same rate until they heard a stop cue (35 cycles). There were three tempos with IOIs of 200, 500, and 750 ms. There were six trials per tempo and tempo order was counterbalanced across participants (see Baer et al., 2015 for more details). Finger movements were recorded using an active, three dimensional motion capture system (Visualeyez VZ3000, Phoenix Technologies, Burnaby, BC, Canada). Two infrared-sensitive cameras tracked the motion of an infrared light emitting diode (LED) that was attached to participants’ right index fingernail using Velcro. The trajectory of the LED was tracked at a sampling rate of 200 Hz and to a spatial resolution of 0.015 mm. The infrared-sensitive cameras were synchronized to the metronome with a National Instruments 6221 Data Acquisition board.

##### Measures

This task was used to measure the ability to produce and maintain an isochronous beat using internal timing processes. Preprocessing and analysis steps were identical to those used in previous studies from this lab (Baer et al., 2013, 2015) which also focused on internal timing processes. Therefore, only taps during the continuation phase were analyzed. Performance for the continuation phase of the task was analyzed to assess the ability to maintain and reproduce an isochronous beat. Mean ITIs were compared to ensure that participants were able to tap out the target interval successfully. In order to characterize long-term drift away from the target interval, the tapping data was linearly detrended. The absolute slope of the detrending line was used as a measure of the magnitude of drift. The variance of the ITIs that remained in the data after detrending was used as a more accurate representation of the cycle-to-cycle tapping variability. According to the Wing and Kristofferson (1973) model, continuation tapping involves two independent processes: an internal timekeeper that acts as a clock generating timing signals, and a motor implementation process which uses input from the time-keeper to accurately time movements. Using this model, tapping variability related to the internal timekeeper and that related to motor implementation were analyzed separately. In addition to these variability measures, use of the motion capture system allowed for analysis of kinematic measures. Smoothness of tapping movement was measured using mean squared jerk (see, Baer et al., 2013). This measure was used to investigate whether between-group differences in tapping performance due to specialized training may be reflected in different kinematic strategies. All measures were averaged over each tempo and compared between groups.

#### The Beat Alignment Perception Test (BAPT)

##### Stimuli and procedure

The BAPT was used to measure the ability to perceive the underlying pulse of a rhythm without requiring a motor response. In this task participants listen to 17 clips of recorded music (average duration = 15.9 s) which have a superimposed computer-generated metronome (1 KHz pure tone, 100 ms duration) that is either in sync or out of sync with the underlying beat. The metronome can be out of sync in one of two ways: stretched (Stretch: at a slower tempo than the music clip) or shifted (Shift: out of phase with the music clip). For each trial participants listened to the stimuli and then were asked to indicate whether the metronome was in sync with the beat or not (Yes or No), and to rate their confidence on a scale of zero to two (0 = just guessing, 1 = pretty sure, and 2 = 100% sure).

Stimuli for the BAPT were created and made available by Iversen and Patel (2008) and the version we used was created by Müllensiefen et al. (2013) for the Goldsmiths Musical Sophistication Index (Gold-MSI) v1.0. Stimuli were presented and responses were recorded with software written in Python (v2.7).

##### Measures

Measures of interest for the BAPT were the proportion of correct yes and no responses as well as the confidence ratings. Percent correct was averaged for each condition (On, stretch and shift). To analyze the confidence ratings, the proportion of responses corresponding to ‘just guessing,’ ‘pretty sure,’ and ‘100% sure’ were averaged over all trials.

#### Working Memory Tasks

In order to assess possible group differences and to examine the involvement of auditory working memory in rhythm abilities, participants were tested on the DS and LNS tasks from the Wechsler Adult Intelligence Scale (WAIS-IV; Wechsler, 2008). In the DS task participants are required to recall strings of numbers and in the LNS task participants are required to recall and mentally manipulate strings of letters and numbers. Tests were scored according to the WAIS manual and age-normed scaled scores were derived.

### Data Analysis

All analyses were conducted using SPSS version 22 (PASW Inc, Chicago, IL, USA). For each task measure a mixed factor repeated-measures analysis of variance (ANOVA) was used with task level (e.g., meter, tempo, or metrical complexity) as within-subject factors and musician type as the between-subjects factor. The Greenhouse–Geisser correction was applied in cases where the assumption of sphericity was violated according to Mauchly’s test. All pairwise and simple comparisons reported below have been corrected for multiple comparisons using the Bonferroni correction. In SPSS, the Bonferroni correction is applied by multiplying the *p*-value by the number of comparisons. In this way, the 0.05 significance threshold can still be used (see The calculation of Bonferroni-adjusted *p*-values, retrieved from http://www-01.ibm.com/support/docview.wss?uid=swg21476685).

In order to examine whether individual experience impacts performance, correlations were performed between the performance measures and five musical experience measures (years of lessons, age of start, current hours of practice, years playing primary instrument, and years playing total). Additionally, we assessed the relationship between auditory working memory and task performance by examining correlations between a combined DS and LNS score and all task measures. Finally, we analyzed correlations between performance measures on all four rhythm tasks. The Benjamini and Hochberg (1995) false discover rate procedure was used to control for multiple correlations. However, as the correlation analysis was exploratory, uncorrected correlation values are reported while values that remain significant following correction are indicated in **Tables 3** and **4**.

**Table 3:** Results of working memory tasks.

	Overall	Drummers	Pianists	Singers	String Players	Non-musicians
Digit span	11.36 (2.93)	10.67 (2.65)	12.36 (2.98)	11.50 (3.78)	12.58 (2.78)	9.86 (1.92)
Letter number sequencing	11.61 (3.04)	11.78 (3.77)	10.55 (1.70)	11.80 (3.52)	13.25 (2.90)	10.79 (2.89)

*Values represent scaled scores. Values outside parentheses are means and values inside parentheses are standard deviations.*

**Table 4:** Results of correlation analysis.

Musical experience	Task (measure)	Pearson r	*p*-value
Years of lessons	RST (ITI deviation)	-0.304	0.053
	RST (% correct)	0.341	0.029
	SCT (motor variability)	-0.370	0.022
Current hours per week	RST (ITI deviation)	-0.426	0.005
	BST (%correct)	0.476	0.002^a^
	BAPT	0.416	0.008

*BAPT, Beat Alignment Perception Test; RST, Rhythm Synchronization Task; BST, Beat Synchronization Task; SCT, Synchronization–Continuation Task; MSJerk, Mean Squared Jerk. ^a^Correlation survived correction for multiple correlations.*

## Results

### Musical Training and Experience

To assess possible differences in training and experience between the musician groups, we used separate one-way ANOVAs for each measure from the MEQ. Only significant or marginally significant differences between groups are reported here (see **Table 1** for all measures). There was a significant effect of age of start [*F*(3,40) = 3.24, *p* = 0.033, η^2^ = 0.208]. None of the between-group comparisons reached significance, however, the drummer group started later on average (*M* = 11.2, *SD* = 3.27), followed by the singers (*M* = 10.7, *SD* = 4.32), string players (*M* = 8.08, *SD* = 2.77), and pianists (*M* = 7.6, *SD* = 2.11). There was a significant main effect of years of lessons [*F*(3,40) = 3.27, *p* = 0.032, η^2^ = 0.212], showing that pianists (*M* = 12.9, *SD* = 2.96) had taken lessons on their primary instrument for significantly longer than drummers (*M* = 5.44, *SD* = 3.61; *p* = 0.030). There were no significant differences between singers (*M* = 11.30, *SD* = 7.97) and string players (*M* = 10.50, *SD* = 5.53) or between these groups and the others. The non-musician group played an instrument for 1.25 years on average (*SD* = 0.94).

### Rhythm Synchronization

**FIGURE 1:**
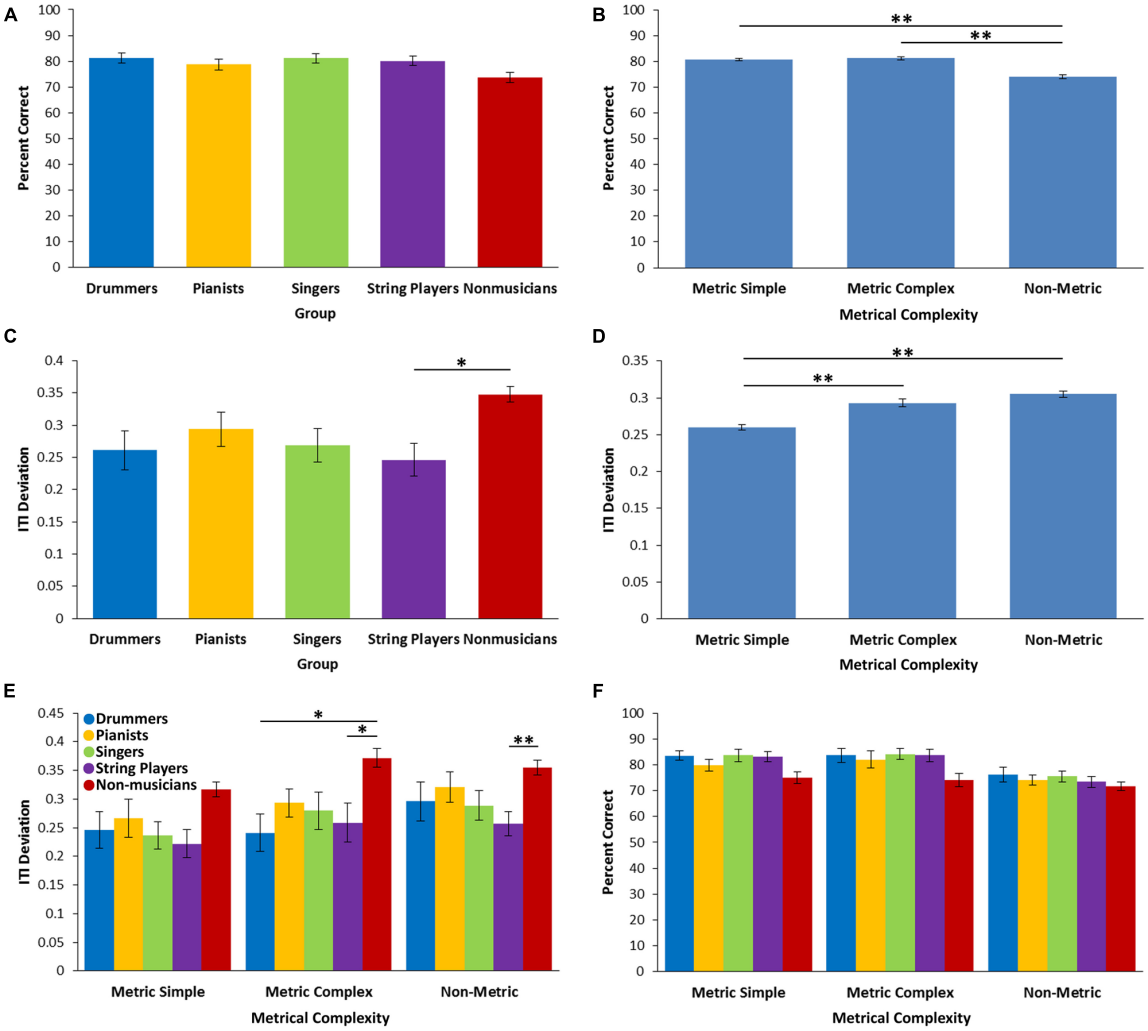
**Performance on the Rhythm Synchronization Task. (A)** Percentage of correct taps across the four musician groups and non-musician control group. **(B)** Percentage of correct taps for each level of metrical complexity averaged over all groups. **(C)** ITI deviations across the four musician groups and non-musician control group. **(D)** ITI deviations for each level of metrical complexity averaged over all groups. **(E)** ITI deviations for each level of metrical complexity for each group. **(F)** Percent correct for each level of metrical complexity for each group. ^∗^*p* < 0.05, ^∗∗^*p* < 0.01.

There was a main effect of metrical complexity for the percent correct measure [*F*(2,100) = 33.93, *p* < 0.001, $\eta_{p}^{2}$ = 0.404], such that participants had a significantly lower proportion of correct taps for the NM rhythms compared to both the MS (*p* < 0.001) and the MC rhythms (*p* < 0.001; see **Figure 1B**). There was a main effect of group [*F*(4,50) = 2.83, *p* = 0.034, η^2^ = 0.185], however, none of the follow-up comparisons survived Bonferroni correction (see **Figure 1A**). There was no metrical complexity by group interaction [*F*(8,100) = 1.27, *p* = 0.267, $\eta_{p}^{2}$ = 0.092; see **Figure 1F**]. This is consistent with the findings of previous studies using the same task (Chen et al., 2008; Bailey and Penhune, 2010, 2012), which show that, based on this global measure, metrical complexity affects performance and that even non-musicians perform adequately.

For the more specific ITI deviation measures, there was a significant effect of metrical complexity [*F*(2,100) = 18.97, *p* < 0.001, $\eta_{p}^{2}$ = 0.275], such that ITI deviation was significantly lower for the MS rhythms compared to both the MC and NM rhythms (*p* < 0.001 for both comparisons). MC and NM rhythms did not differ significantly (*p* = 0.225; see **Figure 1D**). There was a main effect of group [*F*(4,50), = 3.14, *p* = 0.022, η^2^ = 0.20], but no significant metric complexity by group interaction [*F*(8,100) = 1.52, *p* = 0.16, $\eta_{p}^{2}$ = 0.108; see **Figure 1E**]. The main effect of group was driven by a significantly larger ITI deviation in non-musicians compared to string players (*p* = 0.022; see **Figure 1C**). There were no statistically significant differences between musician groups.

### Beat Synchronization

**FIGURE 2:**
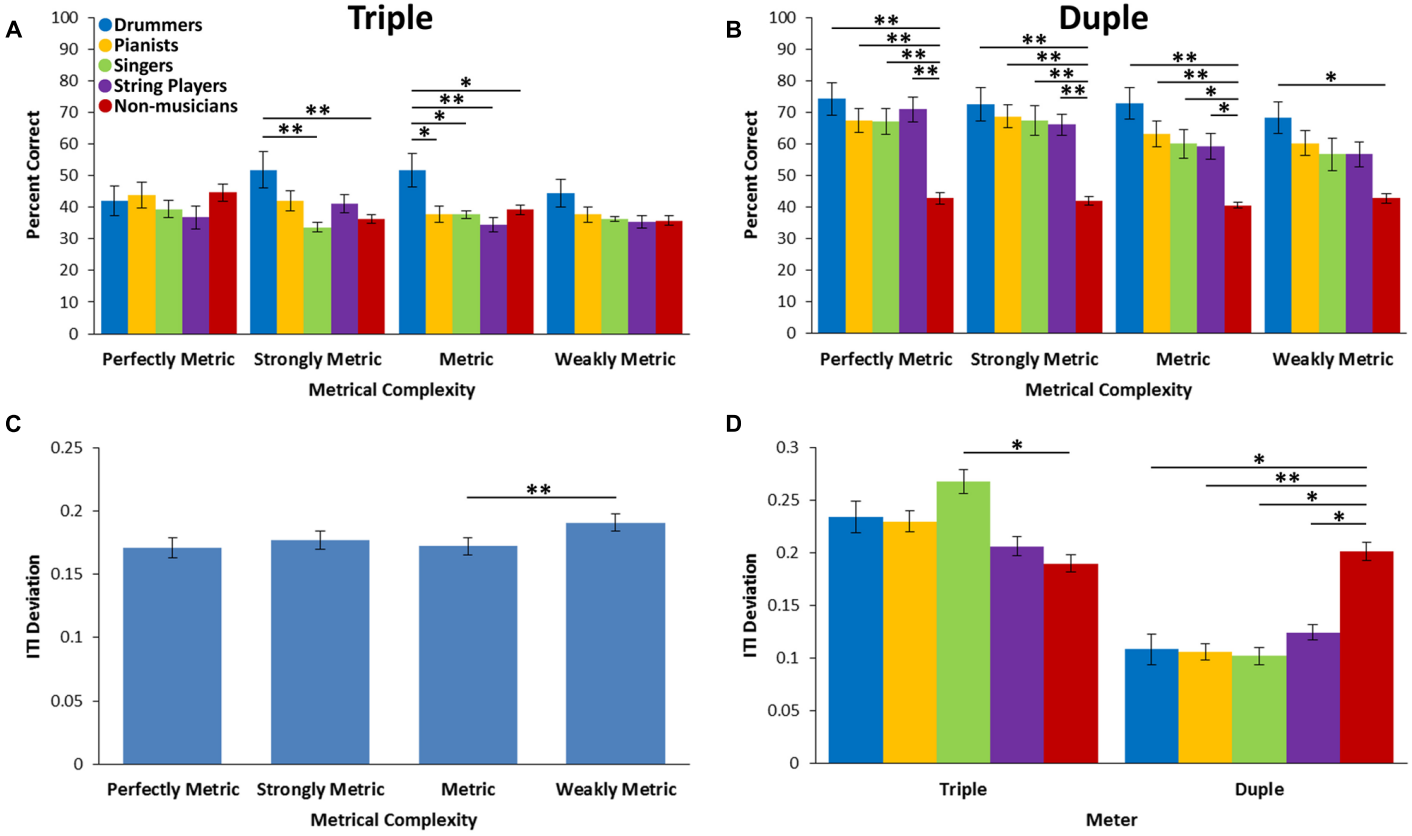
**Performance on the Beat Synchronization Task. (A)** Percentage of correct taps for each group for each level of metrical complexity in the triple meter condition. **(B)** Percentage of correct taps for each group for each level of metrical complexity in the duple meter condition. **(C)** ITI deviations across each level of metrical complexity. **(D)** ITI deviations for each group for triple and duple meter conditions. ^∗^*p* < 0.05, ^∗∗^*p* < 0.01.

Two mixed factorial ANOVAs were performed with percent correct and ITI deviation as dependent variables. Meter (triple and duple), metrical complexity (perfectly metric, strongly metric, metric and weakly metric) and tempo (fast and slow) were included as within-subject factors and musician group as a between-subject factor. For percent correct, there was a main effect of metrical complexity [*F*(3,147) = 11.17, *p* < 0.001, $\eta_{p}^{2}$ = 0.186]. There was also a main effect of meter [*F*(1,49) = 106.09, *p* < 0.001, $\eta_{p}^{2}$ = 0.684], tempo [*F*(1,49) = 27.69, *p* < 0.001, $\eta_{p}^{2}$ = 0.336], and group [*F*(4,49) = 7.94, *p* < 0.001, η^2^ = 0.112]. There was a metrical complexity by tempo interaction [*F*(3,147) = 5.28, *p* = 0.002, $\eta_{p}^{2}$ = 0.097] such that percent correct was higher for slow rhythms compared to fast for all levels of metrical complexity (all *p* < 0.01) except metric (*p* = 0.342). There was also a metrical complexity by meter interaction [*F*(3,147) = 3.04, *p* = 0.031, $\eta_{p}^{2}$ = 0.058] and a three-way interaction with metrical complexity, meter and group [*F*(12,147) = 2.73, *p* = 0.002, $\eta_{p}^{2}$ = 0.182; see **Figures 2A,B**]. No other interactions were significant. Follow-up comparisons showed that for the triple meter, drummers had higher percent correct compared to singers (*p* = 0.003) and non-musicians (*p* = 0.007) for strongly metric rhythms. For metric rhythms in triple meter, drummers had higher percent correct compared to all other groups (Pianists: *p* = 0.015; Singers: *p* = 0.015; String players: *p* = 0.003; Non-musicians: *p* = 0.021; see **Figure 2A**). For duple meter rhythms, non-musicians had lower percent correct compared to all musician groups for perfectly metric, strongly metric and metric rhythms. For weakly metric rhythms, non-musicians had lower percent correct compared to drummers only (*p* = 0.012; see **Figure 2B**). No other comparisons were significant.

For the ITI deviation measure, there was a main effect of metrical complexity [*F*(3,147) = 3.46, *p* = 0.018, $\eta_{p}^{2}$ = 0.066] such that there was higher tapping variability for weakly metric rhythms compared to metric rhythms (*p* = 0.004; see **Figure 2C**). There was a main effect of meter [*F*(1,49) = 39.90, *p* < 0.001, $\eta_{p}^{2}$ = 0.449], a marginally significant main effect of group [*F*(4,49) = 2.56, *p* = 0.05, η^2^ = 0.173] and a meter by group interaction [*F*(4,49) = 4.59, *p* = 0.003, $\eta_{p}^{2}$ = 0.272]. There was no main effect of tempo [*F*(1,49) = 1.74, *p* = 0.193, $\eta_{p}^{2}$ = 0.034]. No other interactions reached significance. Follow-up comparisons showed that in the triple meter, singers showed higher tapping variability than non-musicians (*p* = 0.024). For the duple meter, non-musicians were more variable in their tapping compared to all musician groups (Drummers: *p* = 0.042; Pianists: *p* = 0.005; Singers: *p* = 0.005; String players: *p* = 0.031; see **Figure 2D**).

### Synchronization–Continuation

Following on a large number of studies using the same task (see Repp and Su, 2013 for review), we focused on data from the continuation phase, when internal timing processes, rather than synchronization with external stimuli, were likely to predominate. Mean ITIs were compared across tempi and groups to ensure that participants were able to tap accurately at the three tempi without the aid of a metronome. As expected, there was a highly significant main effect of tempo, with faster rates producing shorter ITIs [*F*(2,100) = 9559.96, *p* < 0.001, $\eta_{p}^{2}$ = 0.995]. Mean rates of for each tempo were 252.23 (*SD* = 9.27), 496.37 (*SD* = 14.68), and 747.12 (*SD* = 33.48). There was no main effect of group [*F*(4,50) = 0.182, *p* = 0.947, η^2^ = 0.014] and no significant tempo by group interaction [*F*(8,100) = 0.835, *p* = 0.530, $\eta_{p}^{2}$ = 0.063]. Together these results show that all groups were able to tap the target intervals successfully, even without the metronome.

**FIGURE 3:**
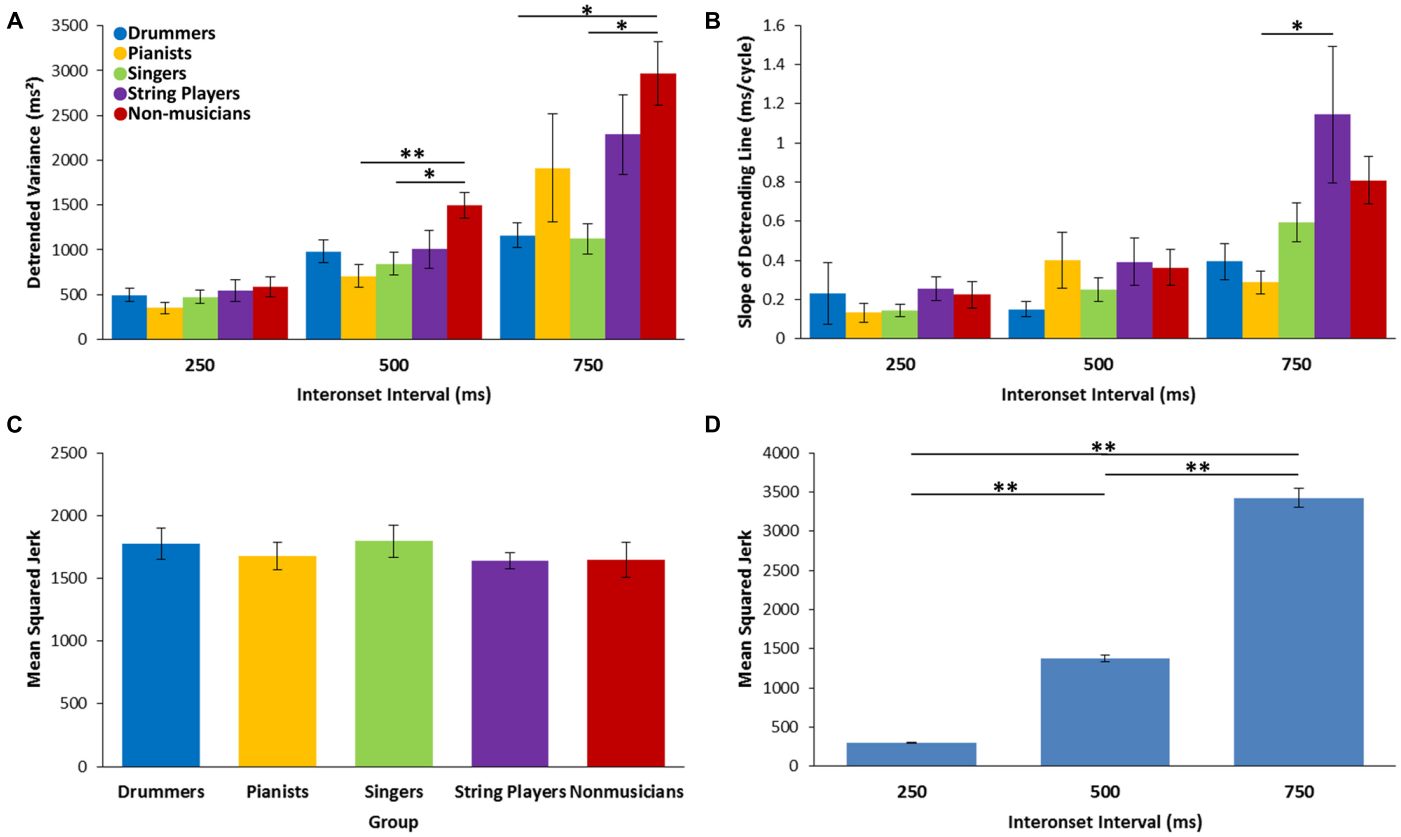
**Variability measures for the Synchronization–Continuation Task. (A)** Detrended variance across the three interonset intervals. **(B)** Slope of the detrending line across the three interonset intervals. **(C)** Mean squared jerk across all groups. **(D)** Mean squared jerk across the three interonset interval averaged over all groups. ^∗^*p* < 0.05, ^∗∗^*p* < 0.01

For detrended variance there was a significant main effect of tempo [*F*(2,100) = 47.01, *p* < 0.001, $\eta_{p}^{2}$ = 0.485], a significant main effect of group [*F*(4,50) = 4.42, *p* < 0.004, η^2^ = 0.261], and a significant tempo by group interaction [*F*(8,100) = 3.06, *p* = 0.019, $\eta_{p}^{2}$ = 0.197]. Follow-up comparisons showed that for the medium tempo (ITI of 500 ms) non-musicians had greater variability than the pianists and singers (*p* = 0.005 and *p* = 0.04, respectively) and that for the slow tempo non-musicians had greater variability than the drummers and the singers (*p* = 0.031 and *p* = 0.019, respectively; see **Figure 3A**). There were no significant differences between musician groups.

The magnitude of long-term drift, as measured by the absolute slope of the detrending line, was compared across groups and tempi. Consistent with previous work (Collier and Ogden, 2004), there was a significant effect of tempo [*F*(2,98) = 16.63, *p* < 0.001, $\eta_{p}^{2}$ = 0.253], such that the magnitude of the slope increased as tempo decreased. There was a main effect of group [*F*(4,49) = 2.93, *p* = 0.03, η^2^ = 0.193] and a tempo by group interaction [*F*(8,98) = 2.22, *p* = 0.054, $\eta_{p}^{2}$ = 0.153; see **Figure 3B**]. Follow-up comparisons showed that string players had a larger absolute slope compared to pianists (*p* = 0.022) at the slow tempo (ITI of 750 ms). There were no other group differences.

As discussed above, mean squared jerk (MSJerk) is a measure of the smoothness of movement, such that smooth movements have low MSJerk. For this measure, there was a main effect of tempo [*F*(2,96) = 565.17, *p* < 0.001, $\eta_{p}^{2}$ = 0.922], showing that MSJerk increased significantly as the tempo decreased, consistent with previous findings (Baer et al., 2013). There was no main effect of group [*F*(4,48) = 0.398, *p* = 0.81, η^2^ = 0.032] and no tempo by group interaction [*F*(8,96) = 0.24, *p* = 0.93, $\eta_{p}^{2}$ = 0.020*;* see **Figures 3C,D**].

**FIGURE 4:**
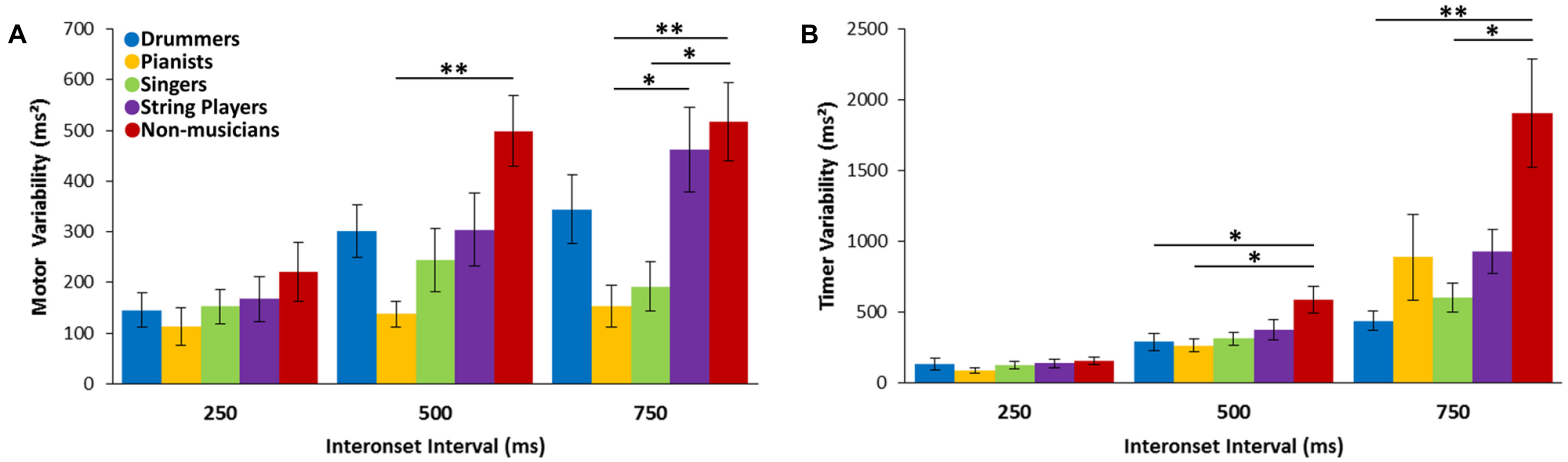
**Wing-Kristofferson variability measures for the Synchronization–Continuation Task. (A)** Motor variability across the three interonset intervals. **(B)** Timer variability across the three interonset intervals. ^∗^*p* < 0.05, ^∗∗^*p* < 0.01.

Tapping variability was split into timer variability and motor variability using the Wing–Kristofferson model. For the timer variability, there was a significant main effect of tempo [*F*(2,96) = 39.96, *p* < 0.001, $\eta_{p}^{2}$ = 0.454], a main effect of group [*F*(4,48) = 7.17, *p* < 0.001, η^2^ = 0.374], and significant tempo by group interaction [*F*(8,96) = 4.16, *p* = 0.004, $\eta_{p}^{2}$ = 0.257]. Follow-up comparisons showed that at the medium tempo, non-musicians showed significantly greater timer variability than drummers (*p* = 0.043) and pianists (*p* = 0.016) but not singers (*p* = 0.066) or string players (*p* = 0.23). For the slow tempo, timer variability was significantly higher in non-musicians compared to drummers (*p* = 0.002) and singers (*p* = 0.005) and near-significant when compared to pianists (*p* = 0.056) and string players (*p* = 0.052). There was no significant between-group differences at the fast tempo (see **Figure 4B**).

For motor variability, there was a significant main effect of tempo [*F*(2,92) = 18.05, *p* < 0.001, $\eta_{p}^{2}$ = 0.282], a main effect of group [*F*(4,46) = 5.44, *p* = 0.001, η^2^ = 0.321] and a tempo by group interaction [*F*(8,92) = 2.32, *p* = 0.026, $\eta_{p}^{2}$ = 0.168]. Follow-up comparisons showed that at the medium tempo non-musicians had greater motor variability than pianists and singers (*p* = 0.002 and *p* = 0.063, respectively). At the slow tempo, string players had greater motor variability than the pianist group (*p* = 0.033) while the non-musicians showed greater variability than the pianists and singer groups (*p* = 0.007 and *p* = 0.02, respectively; see **Figure 4A**).

### Beat Alignment Perception Task

**FIGURE 5:**
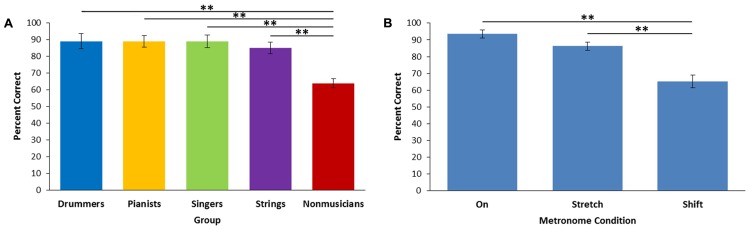
**Performance on the Beat Alignment Perception Test. (A)** Percent of correct responses across groups. **(B)** Percent of correct responses across metronome conditions. Stretch = metronome is slower than beat. Shift = metronome is phase-shifted relative to beat. ^∗^*p* < 0.05, ^∗∗^*p* < 0.01.

One singer and one pianist did not complete the BAPT task. The accuracy of “on” and “off” beat judgments were compared across groups and across the two “off” conditions (stretch and shift; see **Figure 5**). There was a main effect of the on/off variable [*F*(2,98) = 28.29, *p* < 0.001, $\eta_{p}^{2}$ = 0.366], showing that participants were significantly less accurate for the shift condition compared to the on condition (*p* < 0.001) and the stretch condition (*p* < 0.001; see **Figure 5B**). There was a main effect of group [*F*(4,49) = 8.61, *p* < 0.001, η^2^ = 0.413]. Follow-up comparisons showed that non-musicians showed lower accuracy compared to all musician groups across all conditions (*p* < 0.001, for all comparisons; see **Figure 5A**).

In order to test for differences in confidence ratings between groups, proportion of ratings with a value of 0, (just guessing), 1 (pretty sure), and 2 (100% sure) were compared between groups. There was a significant main effect of confidence rating [*F*(2,98) = 122.09, *p* < 0.001, $\eta_{p}^{2}$ = 0.714] and a significant group by confidence rating interaction [*F*(8,98) = 4.62, *p* < 0.001, $\eta_{p}^{2}$ = 0.274]. The main effect of group was not significant [*F*(4,49) = 1.11, *p* = 0.36, η^2^ = 0.083]. Follow up comparisons showed that non-musicians rated their confidence as ‘pretty sure’ for a larger proportion of trials compared to pianists (*p* = 0.009) and drummers (*p* = 0.007) and rated their confidence as ‘100 % sure’ for a smaller proportion of trials compared to pianists (*p* = 0.006) and drummers (*p* = 0.005).

### Working Memory Tasks

Scaled scores on the DS and LNS tasks were compared across the musician and non-musician groups separately. There was no main effect of group for both the DS Task [*F*(4,51) = 2.05, *p* = 0.10, η^2^ = 0.138] and LNS Task [*F*(4,51) = 1.54, *p* = 0.205, $\eta_{p}^{2}$ = 0.108; see **Table 3**].

### Correlations Between Measures of Musical Training, Working Memory, and Task Performance

Analysis of musical experience measures showed significant differences between musician groups in terms of the age at which they started playing their primary instrument and the years of formal training on their primary instrument. Therefore, a correlation analysis was performed to assess whether task performance was correlated with these factors. Years of lessons was significantly correlated with ITI deviation [*r*(39) = –0.30, *p* = 0.053] and percent correct [*r*(39) = 0.34, *p* = 0.029] on the RST as well as with motor variance [*r*(36) = –0.37, *p* = 0.022] on the Synchronization–Continuation task (see **Table 4**).

Because a number of previous studies have shown a relationship between rhythm task performance and working memory (Bailey and Penhune, 2010, 2012; Grahn and Schuit, 2012), we examined correlations between a combined score for the two working memory tasks and the main behavioral measures. The only significant correlation was found with performance on the BAPT [*r*(38) = 0.35, *p* = 0.028 for musicians only; *r*(51) = 0.33, *p* = 0.015 including non-musicians].

Finally, in order to assess how performance on the tasks in our battery related to each other, we examined correlations among the main behavioral measures across tasks for the whole sample. Nearly all task measures correlated significantly with each other and many that were not significant showed a trend towards significance (see **Table 5**).

**Table 5:** Between-task correlations.

	BST (% correct)	BST (ITI deviation)	RST (% correct)	RST (ITI deviation)	BAPT (% correct)	SCT (detrended variance)	SCT (motor variance)	SCT (Timer variance)
BST (% correct)								
BST (ITI deviation)	-0.111							
RST (% correct)	0.409ˆ** a	-0.155						
RST (ITI deviation)	-0.672ˆ**a	0.155	-0.646ˆ** a					
BAPT	0.610ˆ** a	-0.044	0.262	-0.616ˆ** a				
SCT (detrended variance)	-0.343ˆ*a	0.116	-0.528ˆ** a	0.260	-0.349ˆ* a			
SCT (motor variance)	-0.313ˆ*	-0.034	-0.285ˆ*	0.178	-0.296ˆ*	0.710ˆ* a	
SCT (timer variance)	-0.415ˆ** a	0.094	-0.742ˆ** a	0.354ˆ* a	-0.396ˆ** a	0.863ˆ** a	0.451ˆ** a

*^∗^p < 0.05, ^∗∗^p < 0.01, two-tailed. BAPT, Beat Alignment Perception Test; RST, Rhythm Synchronization Task; BST, Beat Synchronization Task; SCT, Synchronization–Continuation Task. ^a^Correlation survived correction for multiple correlations.*

## Discussion

The purpose of this study was to compare the rhythm perception and production abilities of drummers, singers, pianists, string players. A battery of four rhythm and beat-based tasks were used to assess the effects of specific musical training on both higher-level rhythm processing and low-level motor timing abilities.

Existing research had suggested that drummers might outperform other musicians on basic rhythm and interval timing tasks (Krause et al., 2010; Cicchini et al., 2012; Repp et al., 2013). However, our results revealed no significant differences between musician groups for the majority of task measures. This was despite the fact that all musicians were selected to have the majority of their training on the target instrument, had on average more than 10 years of experience on their instrument, and were currently practicing. Generally, musicians showed better performance compared to non-musicians on all rhythm tasks. This suggests that musical training, whether rhythm-focused as in the drummers, or melody-focused as in the singers, improves rhythm perception and production. The only differences between musician groups were found on the BST and SCT.

### Similarities and Differences in Performance Across Tasks

In the BST, drummers had a higher percentage of correct taps compared to all other groups for metric rhythms in triple meter. Additionally, drummers had a higher percentage of correct taps compared to singers and non-musicians for strongly metric rhythms in triple meter. For rhythms in duple meter, all musicians performed equally well and significantly better than non-musicians. This indicates that all musicians were able to use the rhythmic structure of the more common duple meter to find and synchronize to the underlying beat. All musicians had more difficulty synchronizing to the beat of rhythms in triple meter and many performed similarly to non-musicians. The superior performance of drummers for rhythms in triple meter may indicate that the advantage imparted by rhythm-specific training is only evident in the more difficult condition. Another possibility is that drummers are more accustomed to synchronizing with rhythms in triple meter, however, this likely depends less on instrument-specific training than genre-specific training which was not tested here. Differences between musician groups were not seen for the ITI deviation measure. The higher sensitivity of this measure may have increased variability overall which may have obscured more subtle between-group differences.

Continuation tapping in the SCT was used to measure low-level motor timing abilities including the ability to produce and maintain an isochronous beat. No differences between musician groups were found on the detrended variance, MSJerk and timer variance measures. However, pianists were shown to have lower motor variability and less long-term drift, but only compared to string players. Through extensive practice at making precise finger movements, pianists are likely to develop a particularly high level of finger dexterity, possibly explaining their reduced motor variability and drift. In addition, the tapping movement required for this task was very similar to the key-press movements on which pianists train. This is supported by work showing different kinematics in professional pianists compared to amateur pianists (Winges and Furuya, 2015) leading to lower tapping variability compared to non-musicians (Aoki et al., 2005). The lack of other between-group differences in long-term drift indicates that all groups including musicians were able to maintain the target interval over the course of a trial.

The lack of differences between musician groups on the other measures of the SCT may indicate that having a highly developed rhythm framework transfers to low-level tapping abilities. Another possibility is that training on using precise movements to produce music improves the ability to tap accurately, regardless of the specific movements that one is trained in. There were differences in performance on this task between pianists and string players but not for any other groups, a result that cannot be accounted for by either of the above explanations. This suggests that both top-down and bottom-up processes likely interact to determine a musician’s continuation tapping ability.

In the RST, only string players showed lower ITI deviation than the non-musicians. This may be due to high within-group variability on this task. In performing this task, participants are expected to use the metrical structure of the rhythms to better encode and recall the elements of the rhythms, thus facilitating prediction and synchronization (Chen et al., 2008). Enhanced synchronization across all groups for the metric simple and metric complex rhythms compared to the non-metric rhythms, supports the idea that participants were using the metric structure to predict onsets and synchronize finger taps. However, although non-musicians showed lower percent correct and higher ITI deviations, these differences generally did not reach significance (see **Figure 1**). Furthermore, performance on this task was similar to that of musicians and non-musicians tested on this task in previous studies (Chen et al., 2008; Bailey and Penhune, 2010, 2012). This further suggests that the lack of significant differences between musicians and non-musicians is likely due to high variability among the musicians rather than a failure of this particular sample of musicians to perform the task.

For the BAPT, results indicate that perceiving a phase shift relative to the underlying beat of a musical excerpt is more difficult than perceiving a tempo shift for all groups of musicians. Furthermore, musicians were shown to have more sensitive beat perception than non-musicians and non-musicians showed lower confidence in their responses compared to drummers and pianists. However, there were no differences in beat perception between musician groups. This is despite the fact that drummers showed enhanced performance on the BST compared to other the musicians. This difference may due to the importance of movement in beat perception, even for rhythm experts. For example, a recent study showed that percussionists showed improved performance on a beat-based perception task compared to non-percussionists when synchronizing to the beat but were not better in the no-movement condition (Manning and Schutz, 2015). Although, other studies have shown improved performance on basic rhythm and timing tasks (Krause et al., 2010; Cicchini et al., 2012; Repp et al., 2013), it is possible that advantages in higher level beat perception relies on synchronous movement. Another possibility is that this task is not challenging enough to reveal subtle differences in beat perception between the musician groups as the average accuracy was 88%. Between-group differences in the BST were only shown for the more difficult triple meter rhythms. Perhaps with smaller differences between the metronome and the underlying beat, differences in beat perception between musicians would be revealed.

Performance measures on all tasks were highly correlated, indicating that there is strong overlap between perception and production, as well as different rhythm and timing processes. Future work using tasks that account for unique as well as overlapping aspects of rhythm and timing abilities would perhaps detect more subtle differences between musician groups.

The lack of differences between musicians on the tasks requiring tapping, and the lack of advantage for the drummer group in particular, may be due to a discrepancy between the effector and movement type required for these tasks and those used to perform on their instrument. Drummers and percussionists generally perform using drum sticks or mallets and make large, often whole-arm, movements. This discrepancy between effector used in performance and the tasks used here may have reduced the motor timing advantages imparted by drummer’s rhythm-focused training. Continuation tapping with a drumstick leads to lower variability compared to finger tapping in non-musicians (Madison et al., 2013) and percussionists show larger movement-related perceptual timing benefits than non-musicians when tapping with a drumstick (Manning and Schutz, 2015). Furthermore, synchronization with a metronome is less variable when string players synchronize by playing their own instrument compared to finger tapping (Stoklasa et al., 2012). Therefore, motor timing advantages may be specific to the effector and movements that are inherently part of a musician’s training. This is supported by the fact that pianists showed the lowest motor timing variability and long-term drift in the SCT, however, this difference was only significant relative to string players. It has also been suggested that, compared to tapping with a drumstick, finger tapping is more susceptible to small accumulating errors which may increase overall variability and obscuring between-group differences (Madison et al., 2013). Based on this, it would be informative to compare rhythm and timing abilities of musicians using a drumstick and/or their own instruments instead of finger tapping.

Another possible reason for the lack of differences between drummers and the other musicians on these tasks is that the drummer group, although similar to the other musicians in terms of years of experience, had less formal training overall. Although many studies have found differences in synchronization abilities between musicians and non-musicians, two did not find effects related to musical training (e.g., Essens and Povel, 1985; Hove et al., 2010). Other studies did not find differences between musician groups (van Vugt and Tillmann, 2014), or only saw differences in certain contexts (Krause et al., 2010; Carey et al., 2015; Manning and Schutz, 2015). In the study by Krause et al. (2010), drummers had more years of experience than those in the current study (∼15 years vs. ∼12 years in the current study) although they still had less experience than the other musician groups to whom they were compared. Other studies have shown varying results relating to the importance of age of start and years of formal training (Kincaid et al., 2002; Fujii et al., 2009; Bailey and Penhune, 2010, 2012). Therefore, future studies could investigate whether years playing an instrument, age of start or years of formal training contribute differentially to rhythm perception and production abilities in musicians.

### Impact of Musical Experience

There are a number of factors related to music training and experience that have been shown to be related to rhythm perception and production abilities. These include, years of experience, years of formal lessons and age of start, among others (Bailey and Penhune, 2010, 2012; Grahn and Schuit, 2012). Therefore, in this study we attempted to match a number of these potentially confounding variables across our groups. However, specific patterns of experience appeared between the groups that were difficult to control. We were successful in matching the number of years of experience with the primary instrument and the weekly hours of current practice, which did not differ across groups. However, drummers started playing their instrument later and had fewer years of lessons compared to the other musician groups. Age of start was not correlated with any of the task measures, but the number of years of lessons was correlated with percent correct and ITI deviation on the RST, as well as motor variance on the SCT. This is consistent with previous studies using the RST (Bailey and Penhune, 2010, 2012) and may suggest that years of lessons is an important predictor of rhythm abilities. However, this is contrary to a study which showed that tapping stability was correlated with age of start but not years of drum training (Fujii et al., 2009). Therefore, it is possible that fewer years of formal training and/or later age of start in the drummers may have contributed to the lack of differences between them and the other musician groups, despite being matched in terms of years playing their primary instrument.

It is suggested here that musicians with a high level of musical training perform equally well on rhythm tasks, possibly due to extensive knowledge of rhythmic structure in music and strong low-level timing abilities. Therefore, it is possible that testing musicians with intermediate levels of experience would lead to between-group differences. Also, melodies generally contain rhythmic information therefore melody experts such as singers may become rhythm experts as a side effect of their melodic training. Perhaps by comparing musicians based on the type of music they perform (e.g., beat-based vs. not beat-based), differences in rhythmic abilities would emerge. We also show here that non-musicians are generally able to perform tasks that require higher-level rhythm processing, despite the lack of training. This supports the idea that processing even the more abstract aspects of musical rhythm is a skill that is universal among humans.

### Within-Task Comparisons

The lack of differences across musician groups could raise questions as to whether performance or task limitations might affect our results. However, comparison with previous studies using the same tasks, and examination of within-task performance measures indicate that these findings cannot be explained by floor or ceiling effects, or by problems with task manipulations. First, musicians out-performed non-musicians on virtually all task measures, and all musician groups performed in the range of other musicians tested in previous studies. Second, within-task manipulations of metrical complexity, meter and tempo affected performance in predictable ways, consistent with previous studies using the same tasks.

In both the RST and BST, increased metrical complexity led to increased tapping variability and decreased accuracy (Chen et al., 2008; Bailey and Penhune, 2010, 2012; Kung et al., 2013). Similarly, manipulation of meter in the BST and tempo in the SCT showed the expected within-group results (Baer et al., 2013; Kung et al., 2013). Participants were more variable in the BST for rhythms in the triple meter which is consistent with Kung et al. (2013) and was expected as the majority of western music is in duple or quadruple meter. In the SCT, mean ITIs were close to the target intervals showing that participants were able to perform the task successfully. Additionally, tapping variability, long-term drift, jerkiness of movements, as well as motor and timing variability increased as tempo decreased; all consistent with previous work (Repp and Doggett, 2007; Baer et al., 2013, 2015). Likewise, results for the BAPT were consistent with previous research (Iversen and Patel, 2008). Percent correct was higher for the “on” judgments compared to the “off” judgments and participants had more difficulty when the metronome was phase-shifted compared to when it was stretched relative to the beat. Because differences related to within-task factors were consistent with previous research for all groups on all tasks, the lack of between-group differences cannot be attributed to a failure of the task manipulations to alter performance. Finally, these results cannot be attributed to differences in auditory working memory, as no significant differences were found between groups on these tasks.

## Summary and Conclusion

To summarize, we tested drummers, pianists, singers, string players, and a non-musician control group on four rhythm tasks. Overall, musicians performed better than non-musicians on most tasks, however differences between musician groups were not found on a majority of the tasks. Together these results suggest that general musical experience is more important than specialized instrument-specific experience with regards to rhythm perception and production. Only the BST and SCT showed differences between groups. Drummers were better at extracting and synchronizing to the underlying beat of rhythms in the more difficult triple meter condition in the BST. Pianists showed lower motor variability and less drift than string players on the SCT. These results indicate that higher-level and lower-level aspects of rhythm abilities interact in subtle ways such that one may obscure the other when there is a discrepancy between the effector and movements required for the task and those used in training. As only finger tapping tasks were used to measure synchronization and self-paced tapping, drummers only showed higher-level rhythm processing abilities in the most difficult condition of the BST. Conversely, lower-level motor timing advantages were only shown for pianists for the measures that reflect their effector-specific training. The lack of match between training and tasks may have masked differences between groups in all but the most difficult or training-specific conditions. Therefore, musical training improves rhythm abilities in general, whereas more fine-grained, instrument-specific differences are only seen in musicians when task requirements match particular aspects of training.

## Author Contributions

TM was involved with the design, acquisition, analysis, and interpretation of this work as well as writing and revising the manuscript. BG and JT were involved in the acquisition and analysis of the data and contributed to the revising of the manuscript. VP was involved in the conception and design of this work as well as the interpretation of data and revising of the final manuscript.

## Conflict of Interest Statement

The authors declare that the research was conducted in the absence of any commercial or financial relationships that could be construed as a potential conflict of interest.
